# Developing regulatory property of gelatin-tannic acid multilayer films for coating-based nitric oxide gas delivery system

**DOI:** 10.1038/s41598-019-44678-2

**Published:** 2019-06-05

**Authors:** Kyungtae Park, Hyejoong Jeong, Junjira Tanum, Jae-chan Yoo, Jinkee Hong

**Affiliations:** 1 School of Chemical & Biomolecular Engineering, Yonsei University, 50 Yonsei Ro, Seodaemun Gu, Seoul, 03722 Republic of Korea; 2 Biotechnology Research Center, JCBIO Co., LTD & Avison Biomedical Research Center (ABMRC), Yonsei University, Seoul, 03722 Republic of Korea

**Keywords:** Drug delivery, Chemical engineering

## Abstract

To utilize potentials of nitric oxide (NO) gas in anti-bacterial, anticancer, wound healing applications, numerous studies have been conducted to develop a NO delivery system in the past few decades. Even though a coating method and film types are essential to apply in biomedical device coating from previous NO delivery systems, release control from the coating system is still challenging. In this study, we introduced a multilayered polymeric coating system to overcome the uncontrollable NO release kinetics of film systems. We used biocompatible gelatin and tannic acid to construct a rough, porous structured film based on the layer-by-layer self-assembly method. The multilayered polymeric structure facilitated the controlled amount of NO release from (Gel/TA)_*n*_ film and showed burst release in early period owing to their large surface area from the rough, porous structure. We synthesized the proton-responsive NO donor, *N-*diazeniumdiolate (NONOates), into the (Gel/TA)_*n*_ film through a chemical reaction under high pressure NO gas. NO release profile was analyzed by a real-time NO analysis machine (NOA 280i). Then, the NO-releasing (Gel/TA)_*n*_ film was tested its toxicity against human dermal fibroblast cells and bactericidal effects against *Staphylococcus aureus*.

## Introduction

Nitric oxide (NO) is an endogenously produced gas molecule from L-arginine by three nitric oxide synthase (NOS) enzymes in the human body^1,2^. Since the NO gas was verified as a signaling molecule involved in many physiological and pathological processes such as angiogenesis^3–5^, immune response^6,7^, neurotransmission^8,9^, and antibacterial effect^10,11^, many studies have been conducted to develop NO gas delivery systems for biomedical applications. The function of NO in the human body highly depends on its concentration, however, its unstable property makes it difficult to develop a controlled NO delivery system. Therefore, recent studies have focused on developing a controlled release NO delivery system to take advantages of the helpful effect of NO gas in the human body. From this perspective, various types of systems have been reported such as nanoparticles, small molecules that rapidly generate NO, liposomes, the polymer itself, hydrogel, and films^11–15^. Particularly, to apply NO delivery systems in biomedical devices, it is essential to develop a coating or a film system for NO delivery. In previous studies, the sol-gel reaction based coating, self-assembled monolayer, and simple adsorption onto surfaces have been studied as coating systems^14^. However, it is a still challenging to construct a coating system for controlled NO release.

To overcome the limitation of NO delivery coating, we designed the multilayered thin film with a precisely controllable NO donor amount via layer-by-layer (LbL) self-assembly. LbL self-assembly is one of the most widely studied thin film fabrication method because of its versatile process, outstanding properties of controlled drug release and gas capturing, sensing ability^16–20^. LbL technique, as a surface coating method, also can be applied to many types of substrates regardless of the material, shape, and size. Additionally, numerous materials can be used to construct building blocks such as graphene oxide, polypeptides, natural polymers, and synthetic polymers^21–27^. Therefore, it can be an appropriate method with biocompatible materials to apply for biomedical coatings.

Gelatin (Gel) is a natural protein that is highly biocompatible, shows good film-forming ability, and has wide applications in drug delivery, tissue engineering, and hydrogel studies. Gel also has many functional groups that can participate in multiple interactions such as proline and 4-hydroxyproline for hydrophobic interactions, hydrogen bonding, and electrostatic interactions^28^. On the other hand, tannic acid (TA) is a natural polyphenol that can be extracted from plants, thus it is very biocompatible and has been studied for using in many biomedical applications. Additionally, tannic acid has many phenol groups that can bind with proteins through hydrophobic interactions and hydrogen bonding. Because of their biocompatibility and good film-forming property, many studies have been conducted to construct polymeric structures with gelatin and tannic acid^29–31^.

Herein, we used natural materials Gel and TA that emphasized their biocompatible properties in the previous paragraph to develop a polymer-based coating system to improve the controlled release ability of NO delivery by constructing gelatin and tannic acid multilayer (Gel/TA)_*n*._(here, *n* means number of bilayers). We verified the assumption that the film with a roughness, porous structure enabled burst release of NO gas and burst release of NO can have bactericidal effects. We fabricated the multilayer film using the LbL self-assembly technique with different numbers of bilayers, then analyzed the film characteristics measuring the thickness growth tendency and the surface morphology. We confirmed NO donor formation by UV-vis spectrometry and analyzed NO release using a real-time chemiluminescence-based NO analysis device. We tested the applicability of the (Gel/TA)_*n*_ film for biomedical applications via testing toxicity and antibacterial effects against human dermal fibroblast cells (HDF) and *Staphylococcus aureus* (*S. aureus*) which is commonly isolated species in chronic wound via skin infection^32,33^.

## Results and Discussion

### Film growth and characteristics

**Figure 1:**
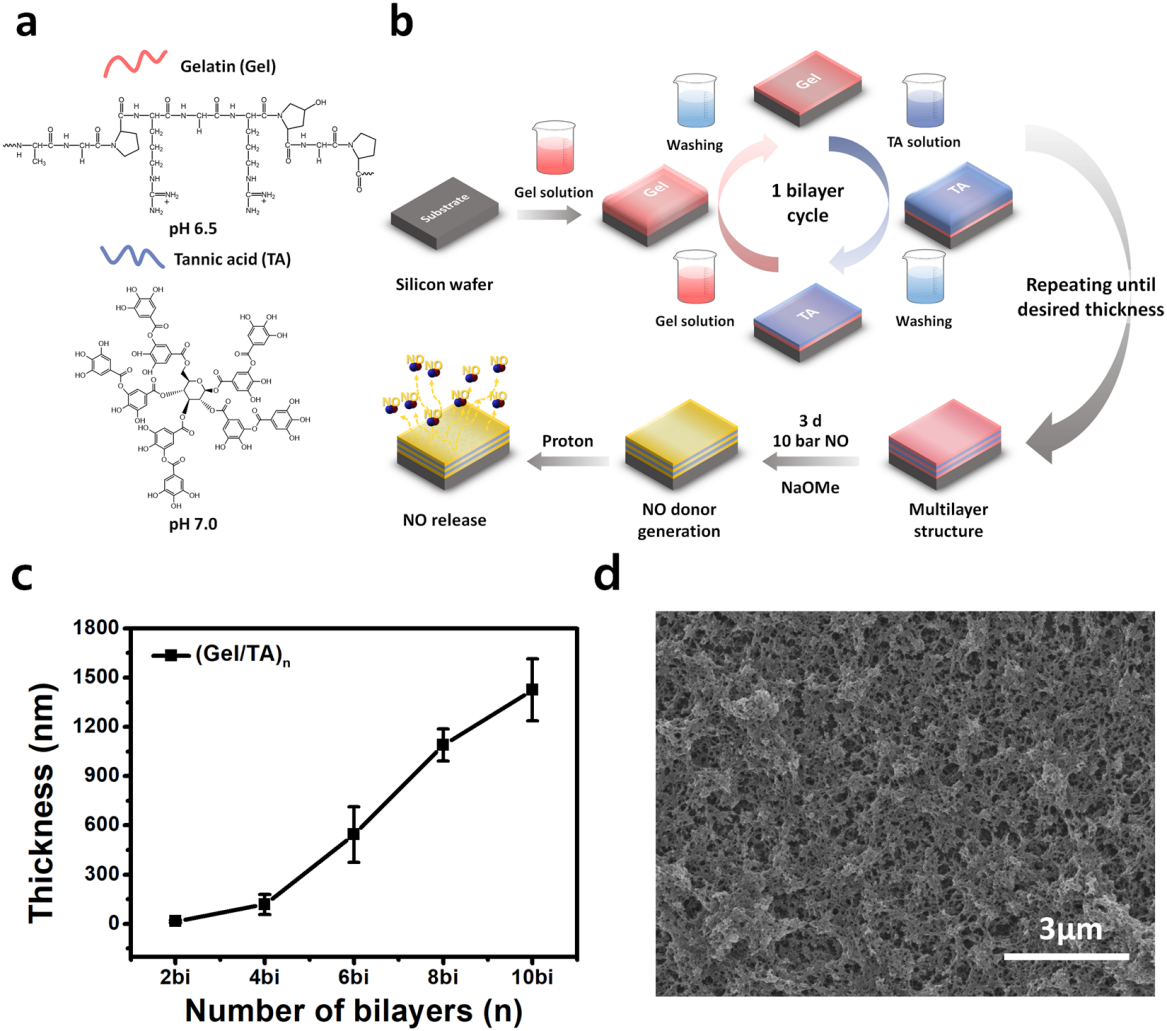
(**a**) Molecular structure and pH condition of film combination and (**b**) schematic illustration about preparation method of gelatin/tannic acid multilayer film for controlled nitric oxide release (**c**) film growth curve measured by profilometry and (**d**) scanning electron microscopy image of 10-bilayer film (Fig. 1 schematic illustration was drawn by K.P.).

**Figure 2:**
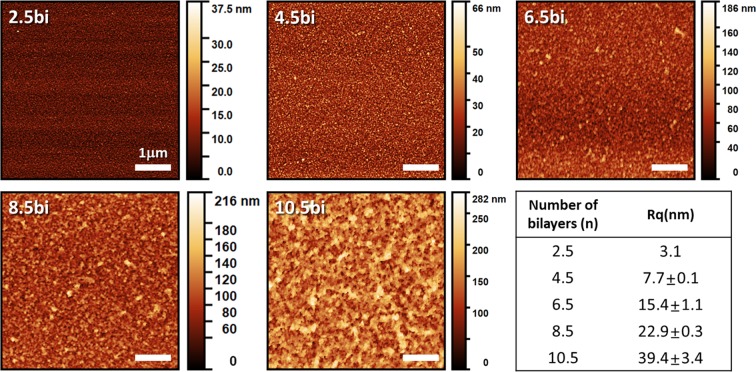
Surface morphology of each film with different numbers of bilayers analyzed by atomic force microscopy.

We fabricated the (Gel/TA)_*n*_ film by the well-known LbL self-assembly method as described in Fig. 1. We attempted to construct the films based on not only hydrophobic interaction, hydrogen bonding but also electrostatic interactions between the neighboring layers to achieve reproducible results. We set the pH value of the Gel solution at 6.5 below its pI value (pH 7–9)^34^ and pH value of TA at 7.0 above its pKa value^29,35^ to induce the ionization of each material for electrostatic interaction. We evaluated film thickness as the number of bilayers increased shown in Fig. 1. The thicknesses of films with 4 bilayers and 10 bilayers were 118.5 nm ± 61.2 nm and 1425.6 nm ± 188.5 nm, respectively. From the thickness results of the film increased regularly with linear growth tendency, we determined the (Gel/TA)_*n*_ film was successfully constructed by LbL assembly. In addition, an approximately 220-nm increase per bilayer was observed from 4 bilayers to 10 bilayers, indicating that the bulk film was constructed. According to many previous studies, the gelatin/polyphenol interaction is based on hydrogen bonding, hydrophobic interactions, and electrostatic interactions^29,35–37^. As the multiple binding sites between the protein and the tannic acid exist, the (Gel/TA)_*n*_ film could diffuse into the neighboring layer during the LbL process^31^. Thus, the drastic increase was valid result considering those multiple interactions and diffusion. The porous and rough structure of the (Gel/TA)_*n*_ film was confirmed from the SEM images shown in Fig. 1d (also see Supplementary Fig. 2) and it is corresponding to the topological images of the film measured by AFM shown in Fig. 2. The porous structure might be formed by a hydrophobic pocket between the hydrophobic side chain of gelatin and aromatic rings of TA, which was stabilized by hydrogen bonding^28^. We measured the root mean square roughness (Rq) using the AFM analysis program as well. Figure 2 shows the increasing tendency of surface roughness with film growth. The 10.5 bilayer film showed 39.4 ± 3.4 nm Rq roughness, which is 10-fold higher than that of the 2.5 bilayer film (3.1 nm). From the film growth data and the surface morphology, we determined that the (Gel/TA)_*n*_ film could be successfully constructed at the certain pH condition and porous structure attributed to the exponential increase. Additionally, we assumed the porous structure and exponential increase of thickness would promote the NO donor generation and their release kinetics.

### Roughness-dependent *N*-diazeniumdiolates (NONOates) synthesis in the Gel/TA film

We selected *N*-diazeniumdiolates (NONOates) as a NO-generating moiety for incorporation into the multilayer film among the many types of NO donor because of a proton-triggered generating mechanism producing two molecules of NO continuously in the physiological condition^32^. We simply synthesized the NO donor into the (Gel/TA)_*n*_ film following the previous works. The synthesis process was carried out under the high pressure of NO gas, called high pressure reaction (HPR), to incorporate the NO donor into the (Gel/TA)_*n*_ films forming the NONOates group at the amine group and amide bond sites of the Gel structure^38,39^. In this chemical reaction, NO gas reacted with the deprotonated amine site. From these reason, we added the sodium methoxide (NaOMe) for promoting deprotonation of the amine group as a catalyst. And it promotes the attack of NO gas into the deprotonated amine and amine groups^39–41^. We measured the film before and after the HPR by UV-vis absorbance to compare the amount of NO donor formation depending on the number of bilayers and its roughness. We hypothesized that the rough, porous structure would make a higher surface area, and cause the proportional increase result of NO donor formation to the thickness. Because of the NONOates formation chemistry based on the deprotonation of amine groups through the sodium methoxide catalyst promoting the nucleophilic attack on nitric oxide^39^, the transformation from amine and amide group to NONOates may have occurred at the interfaces between the solution dissolving sodium methoxide and the films. Thus, the porous, rough structure with the proportionally increased surface area might induce the proportional increase of NO donor to film growth.

**Figure 3:**
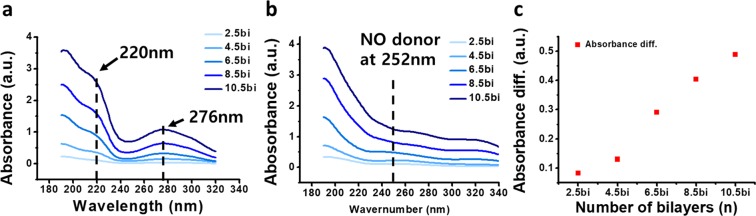
Analysis of nitric oxide donor formation via UV-vis absorbance measurement. (**a**) Absorbance graph before high pressure reaction and (**b**) NO donor peak at 252 nm after high pressure reaction. Absorbance difference for comparing before and after high pressure reaction is depicted in (**c**).

Figure 3 showed the UV-vis absorbance graph comparing before and after NO donor synthesis. Based on previous studies, the NONOates has its specific absorbance peak at the 252 nm wavelength^11,42^. As shown in Fig. 3a, there were specific peaks for TA at 220 and 276 nm^43,44^ and for Gel at approximately 190 nm (normal protein peak). These results correspond to the UV absorbance peak of the Gel and TA solution. (see Supplementary Fig. 1) However, after 3 days of the NONOates synthesis reaction, a specific peak for the NO donor was observed at 252 nm (Fig. 3b). Thus, we confirmed the NONOates was successfully generated in the films. Figure 3c shows the difference in absorbance intensity after the HPR. The peak showed that the amount of NONOates increased proportionally with the thickness. The gradual increasing tendency of peak differences following the number of bilayers increased consistently with the increase in roughness (Fig. 2 roughness table). Consequently, we concluded that as the film grew, the roughness of the films is increased and it affected the amount of NO donor in the film. Therefore, by controlling film roughness and thickness, the loading amount of the NO donor in the film can be controlled.

### NO release profiles

**Figure 4:**
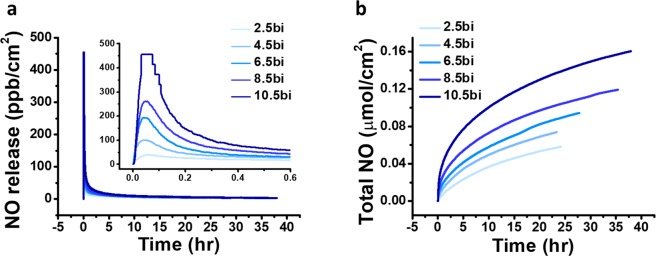
(**a**) Real-time NO release profile measured by nitric oxide analyzer and inset graph showed different time region. (**b**) Accumulated NO release amount depends on the number of bilayers.

Based on this perspective, we analyzed and compared a NO release profile of each film to confirm the correlation between film morphology and NO release control. As the NONOates release NO gas instantly and continuously being triggered by proton, the release profile depends on its hydrated surface area. Because the porous and rough film structure has a higher surface area to volume ratio than a flat surface, they will have higher NO gas release per unit time and area when immersed into the physiological conditions. We evaluated the correlation between film morphology and NO release based on the amount of NONOates quantified from the UV results. We analyzed NO release from each film under mimicked physiological conditions (PBS, pH 7.4, 37 °C). Figure 4 shows the real-time NO release and accumulated NO amount. The (gel/TA)_10.5_ film reached the maximum NO release (454.5 ppb/cm^2^, see Table 1) in 2 min, and the half-life of the total amount released was approximately 5.5 h. Considering that the total duration of NO release was 38.0 h, half of the total NO was released from the film in 15% of the total duration time. It means the (Gel/TA)_*n*_ film has very burst release profile, confirming the correlation between the surface roughness and NO release profile. The (gel/TA)_2.5_ film showed maximum NO release of 38.8 ppb/cm^2^ in 3.8 min to reach it. The (gel/TA)_6.5_ film showed maximum NO release of 193.2 ppb/cm^2^ in 2.7 min to reach it as shown in Table 1. Thus, as film roughness and thickness increased, the maximum amount of NO flux exponentially increased and time consumed for reaching the maximum decreased. The time required to reach the maximum decreased in an inverse proportion from 2.5 to 10.5 bilayers. This can be explained based on previous results and our hypothesis. Increased film roughness produced a larger surface area which could generate more NO donor moieties in the film structure (Fig. 3). Subsequently, the increased film roughness generated NO gas immediately from the interface between the film and PBS solution, resulting in a burst release profile. Considering the fact that NONOates is a proton-responsive NO donor and spontaneously generated NO, the burst release was occurred from the increased surface area to react with protons. Additionally, the total amount of NO released can be controlled depending on the film thickness. As shown in Fig. 4b and Table 1, the total NO released from each film varied from 58.4 to 160.5 nmol/cm^2^. According to the NO release data showing that thicker films generated more NO gas, NO release can be adjusted using the (Gel/TA)_*n*_ film system. In addition, the film showed burst release in the beginning which can be applied for antibacterial coating but also have long release duration and nano-molar range of NO flux that can be applied for promoting cell signaling. Therefore, this the (Gel/TA)_*n*_ system can be a good suggestion to design a NO release coating for biomedical purpose because of their controllable ability and inherent biocompatibilities of its composition materials.

**Table 1:** Summary of NO release profile depending on the number of bilayers.

(Gel/TA)_n_ film	Total NO (nmol·cm^−2^)	t_1/2_ (hr)	[NO]_m_ (ppb·cm^−2^)	t_m_ (min)	t_d_ (hr)
2.5bi	58.4	6.99	38.8	3.8	24.2
4.5bi	73.9	5.69	100.0	2.8	23.4
6.5bi	94.4	6.52	193.2	2.7	27.8
8.5bi	119.2	6.25	261.8	2.8	35.4
10.5bi	160.5	5.56	454.5	2.0	38.0

Total NO: total NO release amount, t_1/2_: half-life of NO release profile, [NO]_m_: maximum NO flux, t_m_: the time consumed to reach the maximum NO flux, t_d_: the duration time of total NO release.

### Film toxicity and antibacterial effect

**Figure 5:**
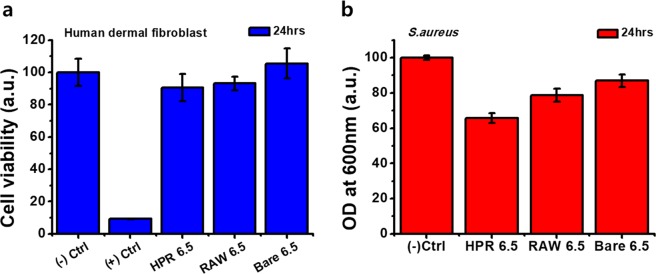
Cytotoxicity test for human dermal fibroblast and anti-bacterial effect against Staphylococcus aureus of (Gel/TA)_6.5_ film. (**a**) Cell viability and (**b**) Bacterial density measured by optical density after treating the sample for 24 h.

To apply this film in biomedical devices and applications, the toxicity test of the film must have preceded. The (Gel/TA)_6.5_ film was used to observe the effects on the human dermal fibroblast (HDF) cells. We prepared three films to investigate the toxicity of NO gas released from the film: bare wafer (BARE 6.5), before high-pressure reaction film (RAW 6.5), and after high-pressure reaction (HPR 6.5) (Fig. 5a). We measured the viability of HDF cells after 1 day of film treatment by MTT assay. The HPR 6.5 sample showed 90% cell viability compared to the negative control group. The RAW 6.5 sample showed 93.2% viability, and thus the NO gas released from HPR 6.5 was not toxic towards HDF cells. Therefore, considering the 420 nm thickness and 94.4 nM of NO release of (Gel/TA)_6.5_ film, this can be applied as a nano-thickness coating such as stent coating for vasodilation or particle coating for cell proliferation^10,45^.

As one of the most widely studied applications of NO in biomedical applications, the antimicrobial effects of nitric oxide have been demonstrated through previous works^10^. Especially, *Staphylococcus aureus*, a common Gram-positive pathogen, has been used as the target bacteria because of its significance that it is the leading cause of skin and soft tissue infections^32,46,47^. The mechanism of the bactericidal effects of NO has arisen from the nitrosative and oxidative species produced by the reaction of NO with oxygen. The reaction produced reactive nitrogen species such as peroxynitrite, nitrogen dioxide, dinitrogen trioxide, and S-nitrosothiols^48^. These species can cause DNA damage, disrupt metabolic enzymes, and consequently induce bactericidal effects^49^. In this study, we evaluated the antibacterial effects of the (Gel/TA)_6.5_ film against *S. aureus*. We measured the optical density to determine the bacteria density in the medium after immersing each film in a multi-well plate for 24 h. We compared the bare wafer (BARE 6.5), before high-pressure reaction film (RAW 6.5), and after high-pressure reaction (HPR 6.5) samples. As shown in Fig. 5b, the HPR 6.5 sample showed 35% decreased bacteria density compared to the negative control. This demonstrated that the (Gel/TA)_6.5_ film has antibacterial effects. Thus, the (Gel/TA)_*n*_ film can be applied as an effective NO delivery system for biomedical device coating and can prevent bacterial infections and cytotoxicity.

## Conclusion

In this study, we developed a multilayer film for a NO gas delivery system by LbL assembly using nature obtained biocompatible materials, gelatin and tannic acid. The film was successfully constructed with 544 nm for 6 bilayers and concisely achieved the different thickness by controlling the number of bilayers. It showed a very rough, porous structure increasing proportionally as film thickness increased. NO donor formation was analyzed by specific UV absorbance peak of NONOates. We verified that the amount of NO donors incorporated in the film is coinciding with the roughness data because the donor formation reaction occurred at the interface of film and the catalyst dissolving solution. The amount of NO released was correlated with the increase in film roughness and the rough film showed rapid and burst release profile. We evaluated the (Gel/TA)_*n*_ film system’s potential in biomedical applications via cell viability testing using HDF cells and antibacterial test against *S. aureus* with (Gel/TA)_6.5_ film. The (Gel/TA)_6.5_ film revealed 90% cell viability and 35% decreased bacteria. In conclusion, the (Gel/TA)_*n*_ polymeric films can be used as a novel NO-releasing coating system with a controlled release profile and high biocompatibility.

## Materials and Methods

### Materials

Gel from porcine skin (type A, gel strength 300), tannic acid (TA), ethanol anhydrous, and methanol anhydrous were purchased from Sigma-Aldrich (St. Louis, MO, USA). Sodium methoxide was from Acros Organics (Geel, Belgium). Silicon wafers were obtained from Namkang Hitech Co., Ltd. (Gyunggi-Do, Korea) and used as substrates.

### Solution preparation

We prepared Gel solution and TA solution at a concentration of 1 mg/mL, which is enough for thermodynamically driven film fabrication of polyelectrolytes^50^. To dissolve the Gel, we heated the Gel solution for 30 min at 60 °C after mixing the Gel powder in deionized water. Subsequently, we stirred the Gel solution for 30 min. We simply prepared the TA solution in deionized water by vigorous mixing for 30 min. The pH of each solution was adjusted using 0.1 and 0.5 M sodium hydroxide solutions. The pH of gelatin solution was 6.5 and that of the TA solution was 7.0.

### Multilayer film fabrication

We used simple immersive method dipping the silicon wafer in polymer solution as described previously^18^. First, the silicon wafer was oxidized with an oxygen plasma treatment machine (CUTE-1B, Femto Science, Somerset, NJ, USA) for cleaning and functionalizing the surface. Next, we dipped the silicon wafer into Gel solution for 10 min followed by rinsing twice for 2 min with pH 6.5 deionized water. The wafer was dipped into TA solution for 10 min followed by rinsing twice with pH 7.0 deionized water for 2 min each. This is the one bilayer fabrication step. We repeated the fabrication steps until achieving films of different thicknesses and we compared their surface morphology and roughness.

### Film characterization

The thickness of the Gel/TA multilayer film was measured with a profilometer (Dektak 150, Veeco, Plainview, NY, USA). The surface morphology of the multilayer film was analyzed by FE-SEM (Carl Zeiss, Oberkochen, Germany). Topographic imaging and root-mean-square roughness measurements were conducted by AFM (NX10, Park Systems, Suwon, Korea). We confirmed *N*-diazeniumdiolate (NO donor) formation in the Gel/TA film by UV-vis (Evolution 300, Thermo Scientific, Waltham, MA, USA).

### *N*-Diazeniumdiolate synthesis into the Gel/TA multilayer film by high-pressure reaction of NO gas

We synthesized *N*-diazeniumdiolates (NONOates) in the Gel/TA multilayer film through a high-pressure reaction (HPR) using NO gas modified previous paper^51^. We prepared anhydrous ethanol and methanol at a 4:1 ratio in a 40-mL vial. We added enough solution to immerse the film. Next, 22.2 µL of sodium methoxide was added in the same molar amount as NONOate formation sites from 10 mg of gelatin. The solution was mixed by vortexing and the Gel/TA multilayer films were placed in a 40-mL glass vial and then placed in a high-pressure reactor (custom-made, Hanwoul Engineering Co., Ltd., Gyeonggi-do, Korea). We purged the reaction chamber with 10 atm of Ar gas after which the chamber was vented immediately. After three rapid purges, we purged the chamber for 10 min with 10 atm of Ar gas and removed the gas three times. The chamber was filled with 10 atm of NO gas and reacted for 3 days. We removed all gas and purged the chamber with 10 atm of Ar gas three times rapidly before removing the samples. The samples were rinsed with pure ethanol and dried under vacuum condition. All experiment process was carried out under room temperature.

### NO release measurement

We analyzed the real-time NO release profile for the multilayer film using an NO analyzer (Sievers NOA 280i, GE Analytical Instruments, Little Chalfont, UK) in phosphate-buffered saline (PBS) at pH 7.4 and 37 °C. Ar gas was used as a carrier gas to move the generated NO gas. We measured the NO release profile to below 10 ppb.

### Cell viability test

We investigated cell viability by the MTT (3-(4,5-dimethylthiazole-2-yl)-2,5-diphenyl tetrazoliumbromide, M2128, Sigma) assay. We seeded human dermal fibroblasts (HDF) into a 24-well plate with 1 × 10^4^ cells in each well and cultured the cells in HDF growth medium (Dulbecco’s Modified Eagle’s Medium containing 10% vol fetal bovine serum and 1% vol penicillin-streptomycin) at 37 °C and 5% of CO_2_ overnight (n = 3). After the cells adhered, the (Gel/TA)_6.5_ film was placed in the wells and cultured for 24 h. The samples were then removed and the culture medium was replaced with a 10% MTT solution. After 2 h, the culture medium was removed and MTT formazan was dissolved by DMSO. The absorbance of each well was measured at 570 nm using a plate reader.

### Antibacterial test

We purchased *Staphylococcus aureus* cells from the American Type Culture Collection (Manassas, VA, USA). *Staphylococcus aureus* was cultured in tryptic soy broth (soybean-casein digest media; BD Biosciences, Franklin Lakes, NJ, USA) at 37 °C under aerobic conditions. We used bacterial strains at an optical density (OD, measured at 600 nm) of 0.58. We placed the (Gel/TA)_6.5_ multilayer films (before (RAW 6.5) and after (HPR 6.5) and the bare silicon wafer (BARE) in a 24-well plate with tryptic soy broth medium and *S. aureus* for 24 h. Following the appropriate culture time, the wafers were removed. Next, we measured the OD at 600 nm with a plate reader to analyze bacterial growth.

## Supplementary information

 UV-vis absorbance data of gelatin and tannic acid solution and cross-section SEM image are accompanied for this paper.
