# Supplementary material for: Developing regulatory property of gelatin-tannic acid multilayer films for coating-based nitric oxide gas delivery system
Source: Sci Rep. 2019 Jun 5;9:8308. doi: 10.1038/s41598-019-44678-2 (PMC6549184; doi:10.1038/s41598-019-44678-2)
Supplement: Supplementary file 1 — UV-vis absorbance data of gelatin and tannic acid solution and cross-section SEM image are accompanied for this paper. [file 41598_2019_44678_MOESM1_ESM.docx]

**Developing regulatory property of gelatin-tannic acid multilayer films for coating-based nitric oxide gas delivery system**

Kyungtae Park^a^, Hyejoong Jeong^a^, Junjira Tanum^a^, Jae-Chan Yoo^b^ and Jinkee Hong^a, *^

^a^ School of Chemical & Biomolecular Engineering, Yonsei University, 50 Yonsei Ro, Seodaemun Gu, Seoul 038722, Republic of Korea

^b^ Biotechnology Research Center, JCBIO Co., LTD & Avison Biomedical Research Center (ABMAC), Yonsei University, Seoul 03722, Republic of Korea

E-mail: ^a^ jinkee.hong@yonsei.ac.kr

*Corresponding author

**Supplementary information.**

**
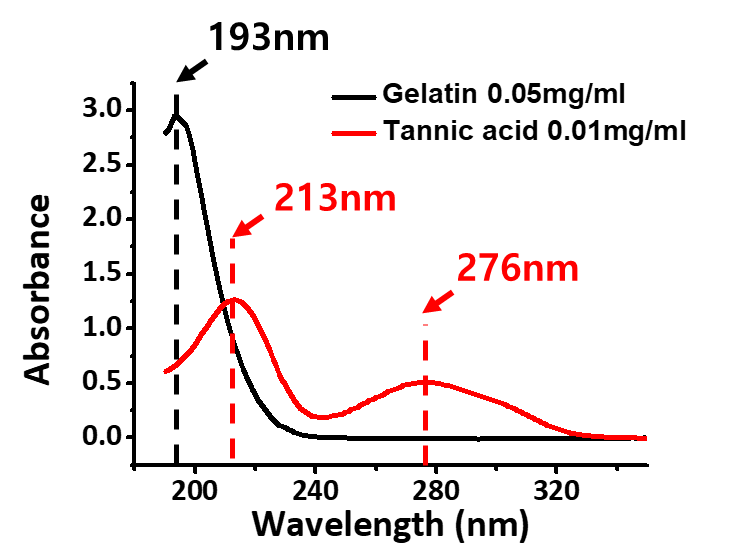
**


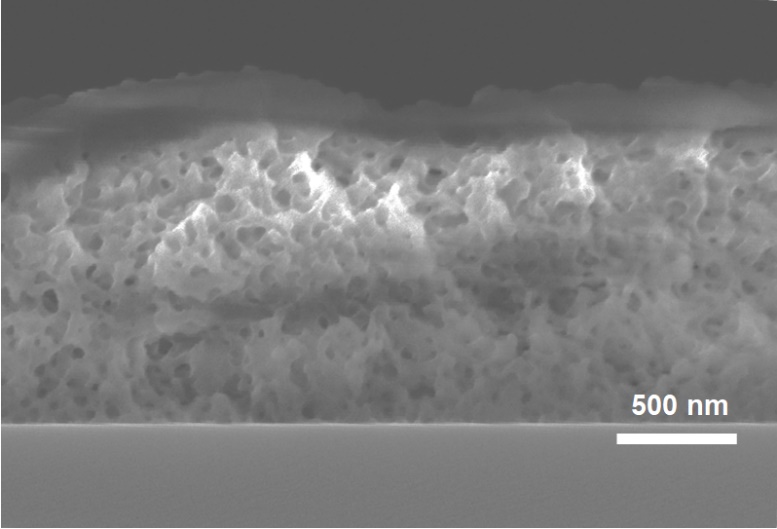
**Supporting figure 1** UV-vis absorbance peak of gelatin and tannic acid solution. Red line indicates the tannic acid solution (concentration: 0.01 mg/mL) and black line indicates the gelatin solution (concentration: 0.05 mg/mL).

**Supporting figure 2** Cross-section scanning electron microscopy image of 10.5-bilayer gelatin tannic acid film.
